# Stop helping pathogens: engineering plant susceptibility genes for durable resistance

**DOI:** 10.1016/j.copbio.2021.05.005

**Published:** 2021-06-19

**Authors:** Hernan Garcia-Ruiz, Boris Szurek, Guido Van den Ackerveken

**Affiliations:** 1 Department of Plant Pathology, Nebraska Center for Virology, University of Nebraska-Lincoln, Lincoln, NE 68503, USA; 2 PHIM Plant Health Institute, Univ Montpellier, IRD, CIRAD, INRAE, Institut Agro, Montpellier, France; 3 Plant–Microbe Interactions, Department of Biology, Utrecht University, Padualaan 8, 3584 CH Utrecht, Netherlands

## Abstract

Alternatives to protect crops against diseases are desperately needed to secure world food production and make agriculture more sustainable. Genetic resistance to pathogens utilized so far is mostly based on single dominant resistance genes that mediate specific recognition of invaders and that is often rapidly broken by pathogen variants. Perturbation of plant susceptibility (*S*) genes offers an alternative providing plants with recessive resistance that is proposed to be more durable. *S* genes enable the establishment of plant disease, and their inactivation provides opportunities for resistance breeding of crops. However, loss of *S* gene function can have pleiotropic effects. Developments in genome editing technology promise to provide powerful methods to precisely interfere with crop *S* gene functions and reduce tradeoffs.

## Introduction

Plant susceptibility (*S*) genes enable successful pathogen infection, and their perturbation can render plants resistant to pathogens [1]. Engineering *S* genes, for example, by genome editing, has great potential for the generation of disease resistant crops.

Susceptibility to pathogens is actively facilitated by the host plant [2]. At the same time, plant immune responses are actively suppressed by pathogen effectors, proteins that interfere with host processes and promote susceptibility, and by intrinsic plant immunity regulators, thereby favoring disease development. Recessive *S* gene alleles, conferring resistance, have been identified following mutagenesis or as natural variants, for example, the *mlo* allele confering resistance to powdery mildew, the rice *xa13* allele providing resistance to *Xanthomonas* bacteria, and *eIF4* confering potyvirus resistance (Box 1). More recently, *S* genes were found through pathogen effectors and the host targets they manipulate.

**Figure 1:**
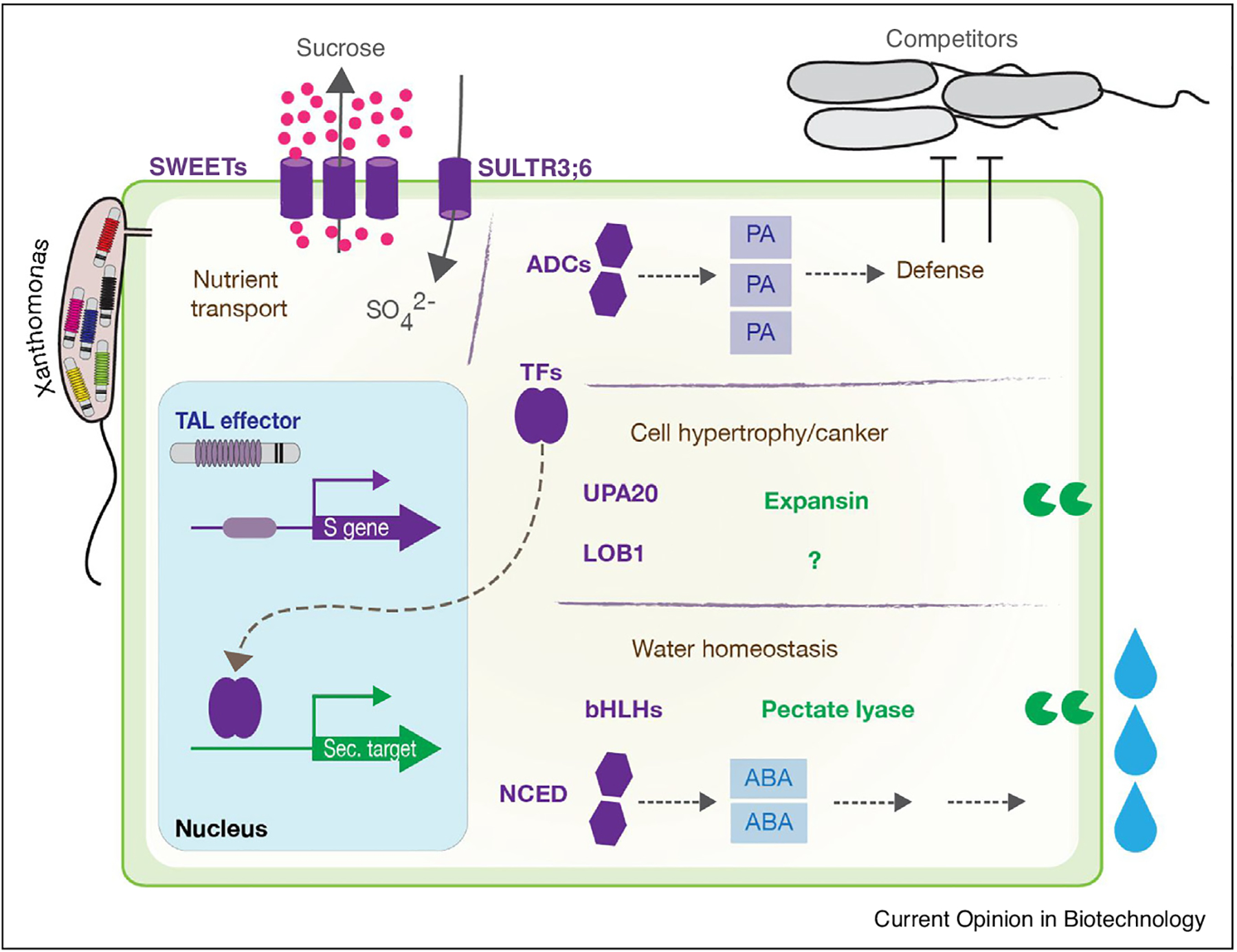
Activities of S genes targeted by TAL effectors. Our in-depth understanding of the molecular mechanisms underlying TAL effectors (TALe) action revolutionized the quest for their targets in planta. Because TALes act as bona fide eukaryotic transcription factors which DNA-binding sites are highly predictable, transcriptomic approaches combined to *in silico* target promoter search allows for rapid identification of their target gene candidates. To such an extent that nearly 10 classes of S genes have been discovered since the elucidation of the TAL code in 2009 [49,50]. Their function is quite diverse, ranging from sucrose (SWEET) and sulfate transporters, enzymes involved in the biosynthesis pathway of various compounds such as polyamines (arginine decarboxylases), ABA (9-cis-epoxycarotenoid dioxygenase) or even small RNAs (the methyltransferase Hen1), to different types of transcription factors (LOB, bHLH, bZIP, ERF) involved in the control of various phenotypes such as host cell enlargement, pustule formation, watersoaking, and so on…It is expected that other categories of S genes will be discovered as novel TAL effectors with major or even moderate virulence functions are characterized. The potential is high because the majority of *Xanthomonas* species rely on TALes to infect their host and only the *S* genes corresponding to 7 pathosystems have been investigated today when there are at least fifty species or pathovars of *Xanthomonas* with unique features that remain to be investigated. This figure gives an overview of the most relevant *S* gene categories targeted by TALes and for which a function is described. Text in brown refers to the types of activities conferred by *S* genes. Primary and secondary targets are shown in purple and green (text, shape), respectively. Abbreviations: SWEET, Sugars Will Eventually Be Exported Transporter; SULTR, sulfate transporter; ADCs, arginine decarboxylases; PA, polyamines; TFs, transcription factors; UPA, upregulated by AvrBs3; LOB1, lateral organ boundaries 1; ABA, abscisic acid; bHLH, basic helix-loop-helix; NCED, 9-cis-epoxycarotenoid dioxygenase. Forms: cylinder, nutrient transporter; hexagon, biosynthetic pathway enzyme; two-ovoid, transcription factor; Pacman-like, cell wall-modifying proteins.

**Figure 2:**
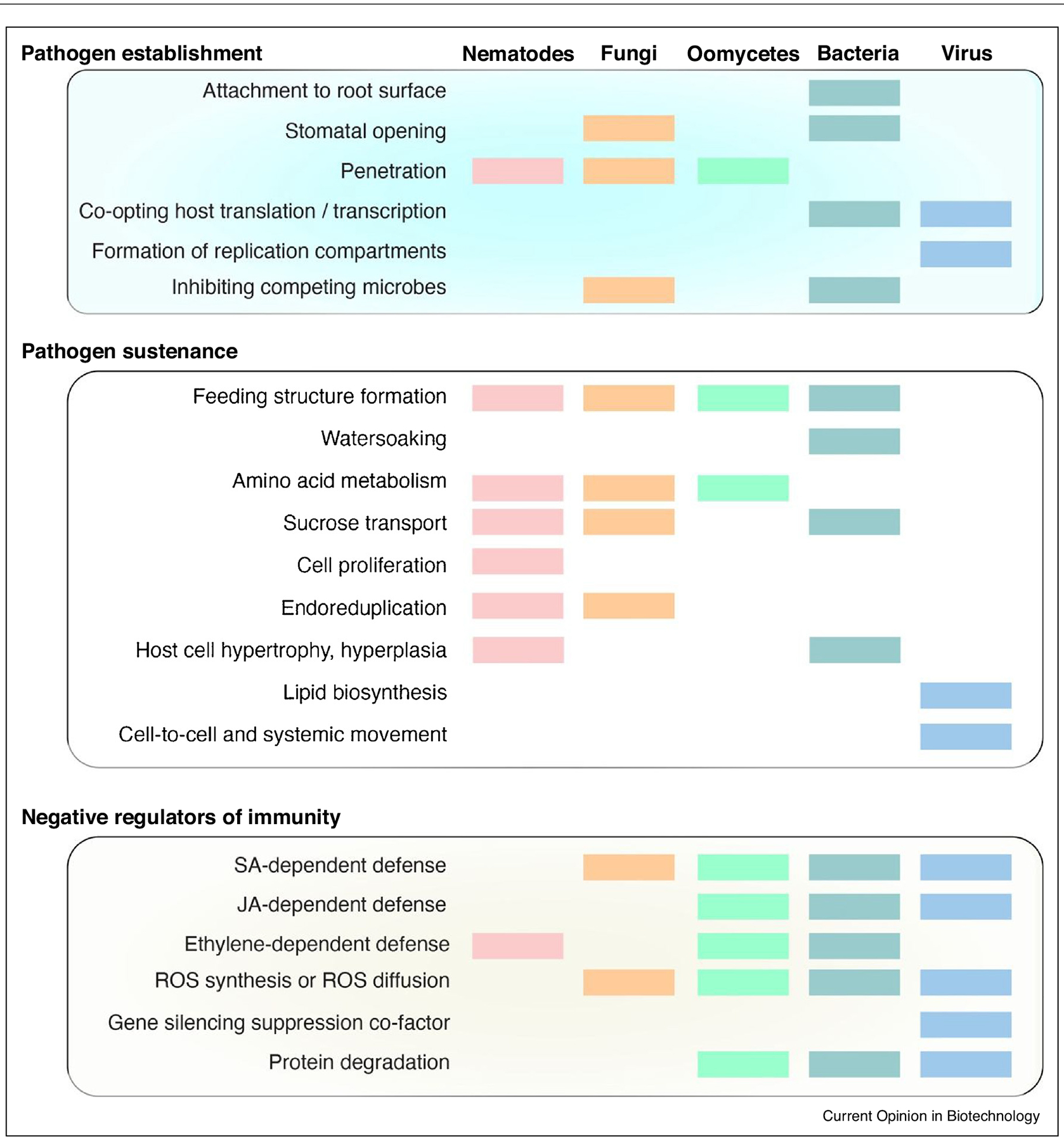
Categories of S genes based on the mechanistic activity. Conservation of S genes across pathogen groups is color coded.

The diversity of *S* genes is strikingly illustrated by the targets of bacterial Transcription Activator-Like effectors (TALes) that are active inside host cells to activate a plethora of *S* genes that support bacterial infection (Figure 1). Known plant *S* genes contribute to pathogen establishment, pathogen sustenance, or may be negative regulator of host immunity (Figure 2). Here, we present recent advances in understanding *S* gene functions, pleiotropic effects of their mutation, and opportunities and challenges of *S* gene engineering to generate disease resistant crops.

While necrotrophic pathogens also produce effectors to modulate host susceptibility gene products [3], their cell death inducing activities are very different from mode of actions observed in interactions with biotrophic pathogens. In our review we focus on *S* genes that are needed for biotrophic interactions.

## Pathogen establishment

Successful infection and ensuing disease development require that pathogens are accommodated by the plant host, creating favourable niches for growth and further spread.

### Accommodation

*S* genes have recently been identified that were previously known to be important for the accommodation of beneficial microbes, for example, arbuscular mycorrhiza that form tree-shaped haustoria for nutrient exchange in root cells [4]. Haustoria are also formed by many fungal and oomycete pathogens by invagination of the host cell membrane. Therefore, it was tested if symbiotic mutants are also affected in their susceptibility to pathogens. Mutation of the Medicago *API* and *RAD1* genes also perturbed susceptibility to the root infecting *Phytophthora palmivora* [5,6^•^]. In Arabidopsis, mutation of several orthologs of legume symbiosis genes resulted in reduced susceptibility to downy mildew [4]. Common symbiosis genes can thus act as *S* genes in pathogenic interactions.

Similarly, parasitism of cyst and root knot nematodes is dependent on modifications of plant cells. The formed syncytia function as hypermetabolic nematode feeding sites, and requires nematode-dependent cytokinin signaling, mediated by histidine kinase receptors. Arabidopsis mutants lacking receptors AHK2 and AHK3 are less susceptible to cyst (*Heterodera schachtii*) and root knot nematodes (*Meloidogyne incognita*) [7,8].

### Enabling virus infection

*S* genes are needed at all stages of viral infection: virion disassembly, viral RNA translation, replication complex formation, genome replication, transcription, cell-to-cell movement, systemic movement, and virion formation. An example is heat shock protein 70-2 (Hsc70-2) which physically interacts with beet black scorch virus (BBSV) protein p23 during the formation of virus replication compartments in the endoplasmic reticulum. In the absence of Hsc70-2, virus replication complexes are not formed. Overexpression and downregulation of Hsc70-2 enhanced and drastically reduced BBSV accumulation in plants, respectively [9].

### Establishing a favourable environment

Several bacterial pathogens were recently shown to create an aqueous environment in their host. *Xanthomonas gardneri* indirectly activates a pectate lyase in tomato [10] and *Xanthomonas translucens* stimulates the ABA biosynthetic pathway in wheat [11^•^], both resulting in induced watersoaking which is suggested to promote bacterial multiplication and/or spread. The activation of these pathways by TALes is shown in Figure 1.

## Sustenance of pathogens

Once infections are established, pathogens need continued provision of nutrients and cellular host factors to sustain colonization of the host.

### Pathogen feeding

Sugar transporters contribute to pathogen proliferation. Several bacterial species hijack host nutrient secretion systems for efficient pathogen reproduction in planta, as illustrated by the SWEET sucrose efflux exporters in rice. Their transcriptional induction by Xanthomonas TAL effectors is crucial for disease development [12]. The role of SWEET sugar transporters in susceptibility seems to be conserved in other hosts, such as cotton and cassava, and in infections with TALe-lacking pathogens, for example, *Pseudomonas syringae* [13], a clubroot-causing fungus [14] and root knot nematodes [15]. The exploitation of SWEET transporters by such a diverse array of pathogens allows to define them as susceptibility hubs. Indeed, perturbation of three major *SWEET* susceptibility genes in rice elite mega varieties by multi-editing of 6 TALe binding-sites within the promoter leads to broad spectrum resistance against the bacterial blight pathogen *Xanthomonas oryzae* pv. oryzae [16^••^].

Several other sugar transporters, or dominant gain-of-function alleles thereof, are not *S* genes but contribute to resistance. This is illustrated by the STP13 family of hexose transporters [12].

*Ralstonia solanacearum* hijacks plant host metabolism for the biosynthesis of gamma-aminobutyric acid (GABA) to support its growth. Inside plant cells, the RipI effector promotes the biochemical activity of glutamate decarboxylases (GADs) and enhances GABA production to support bacterial nutrition [17].

### Viral movement

Plant viruses move cell-to-cell through plasmodesmata. The endoplasmic reticulum (ER) is interconnected among cells via desmotubules. Tomato spotted wilt virus protein NSm associates with the ER and mediates virus cell-to-cell movement. The Arabidopsis *rdr3* mutant, with a non-branched ER network, shows a clear delay in viral cell-to-cell movement, despite efficient replication [18]. This is just one example of plant *S* genes enabling viral spread and sustenance that could be perturbed to engineer virus resistance.

## Negative regulation of plant immunity

An important group of *S* genes encodes negative regulators of immunity that plants use to fine tune defense responses and limit tradeoffs [19]. Mutants in such *S* genes show enhanced resistance, often to a broader range of pathogens. Some negative regulators are targeted by pathogen effectors to stimulate their suppressive effect on plant immunity.

### Endogenous negative regulators of immunity

Barley *Mildew locus o* (*Mlo)* encodes a membrane protein that is needed for negative regulation of immunity (Box 1). In Arabidopsis, the *mlo2 mlo6 mlo12* triple mutant shows elevated and more rapid accumulation of defense-related transcripts in response to powdery mildew infection [20]. However, mutation of known defense-related and metabolic genes did not abolish *mlo* resistance to powdery mildew, suggesting it is caused by an unknown mechanism [21]. Interestingly, *MLO* silencing in pepper also conferred resistance to the bacterium *R. solanacearum* [22] and in cucumber to the fungus *Corynespora cassiicola* [23].

Powdery mildew resistance is also obtained by mutation of *ENHANCED DISEASE RESISTANCE1* (*EDR1*). The Arabidopsis EDR1 protein kinase was recently described to interfere with the heteromeric association of the immune regulators EDS1 and PAD4. Mutation of *EDR1* is thought to enhance the formation of an EDS1-PAD4 heterodimer that is needed to activate defense and resistance [24]. In hexaploid wheat, powdery mildew resistance was obtained by genome editing 3 homeologous *EDR1* genes [25].

*DOWNY MILDEW RESISTANT 6* (*DMR6*) is an *S* gene acting on a broader range of biotrophic pathogens [26]. It encodes an oxygenase that hydroxylates salicylic acid (SA), thereby downregulating defences [26,27]. Genome editing of *DMR6* in tomato resulted in plants showing enhanced resistance to the bacterial pathogen *Xanthomonas* [28], and in potato to the late blight pathogen *Phytophthora infestans* [29]. Interestingly, the rice *DMR6* ortholog *Os03g03034*, is induced by *Xoo* and *Xoc* TALes, suggesting pathogens can transcriptionally activate plant negative regulators to enhance susceptibility [30].

Metabolism can also negatively affect plant immunity. In a collection of wheat lines rust resistance was correlated to differential expression of amino acid metabolism genes. Disruption of the associated branched-chain aminotransferase 1 gene (*TaBCAT1*) in wheat resulted in reduced susceptibility to yellow and stem rust infection and was associated with increased SA levels and defense gene expression [31^••^].

### Effector-mediated negative regulation

Effector-mediated manipulation of the stability of negative regulators of immunity is a way by which pathogens enhance susceptibility. Grape VvWRKY40 is proposed to be stabilized by effector PvRXLR111 during *Plasmopara viticola* infection. Silencing of the orthologous gene in *Nicotiana benthamiana* resulted in reduced susceptibility to the oomycete *Phytophthora capsici* [32].

In potato, several *S* genes have been identified through effectors of the late blight pathogen *P. infestans*. Two recent examples are *StVIK*, encoding a putative MAP3K [33], and *NRL1* (*NPH3/RPT2-LIKE1*) encoding for a putative substrate adaptor component of a CULLIN3 ubiquitin E3 ligase. Silencing of the *S* gene *NRL1*, resulted in stabilization of SWAP70 that in turn positively regulated plant immunity [34].

Also viruses can stimulate negative regulation of immune response, for example, gene silencing that is critical for plant antiviral immunity [35]. Begomovirus infection can stimulate accumulation of calmodulin-like protein (rgs-CaM) that acts as a negative regulator of gene silencing. rgs-CaM reduces RDR6 transcription and directs SGS3 to degradation [36], thereby diminishing gene silencing amplification and enhancing virus accumulation.

## Pleiotropy (tradeoffs)

As *S* genes often participate in multiple pathways, including development, their inactivation may result in reduced fitness [18], perturbed interaction with beneficial microbes [35], or gain of susceptibility to other pathogens.

### Reduced plant fitness

Many loss-of-susceptibility mutants show growth reduction and physiological tradeoffs, for example, early senescence in the barley *mlo* mutant [20]. Accumulation of the defense hormone salicylic acid (SA) is often causing growth-immunity tradeoffs [19]. However, reduction of SA does not always restore growth, for example, in the Arabidopsis *dnd2* mutant in which growth appears to be impacted by altered auxin and abscisic acid levels [37].

### Perturbed beneficial interactions

Loss-of-susceptibility mutants risk to also be impaired in interactions with beneficial microbes. Although resistant to powdery mildew, barley *mlo* mutants were compromised in colonization by the root endophyte *Serendipita indica*, while colonization by mycorrhizal fungus *Funnelisformis mossseae* was higher than in wild type plants [38]. Also, many accommodation mutants, as discussed earlier, show impaired interactions with beneficials, for example, in Medicago *rad1* mutants that have strongly reduced colonization by arbuscular mycorrhiza fungi [5].

### Increased susceptibility to other pathogens

Resistance to one group of pathogens can sometimes lead to an increase in susceptibility to others, for example, powdery mildew resistant wheat and barley *mlo* mutants are more susceptibility to blast caused by *Magnaporthe* [39]. Contrasting effects on viruses were detected after knocking down Glyceraldehyde 3-phosphate dehydrogenase (GAPDH) in *N. benthamiana. GAPDH* silencing leads to loss of susceptibility to tomato bushy stunt virus (TBSV), but does not affect tobacco mosaic virus (TMV) [40]. In contrast, *GAPDH* silencing led to enhanced susceptibility to bamboo mosaic virus (BaMV) [41]. Similarly, knock out of *eIF4E1* in *Arabidopsis thaliana* resulted in resistance to clover yellow vain virus (CIYVV), but hypersusceptibility to turnip mosaic virus (TuMV) [42].

Fortunately, there are also cases where no antagonistic pleiotropy is observed, for example, in rice *SWEET* poly mutants that showed no changes in agronomic performance when tested in microfield trials [16^••^].

## Engineering crop *S* genes

In many crops, *S* genes have been inactivated by classical mutation breeding, that is, identifying defective alleles from mutagenized plant lines. Also, pleiotropic effects of *S* gene inactivation have been minimized by conventional breeding using suitable genotypes, or by selecting mild *S* alleles. The emergence of genome editing technologies over the last decade has sparked the possibilities to engineer changes in crop genomes and thereby the broad utilization of *S* genes [43]. Genome editing can be deployed to fine-tune *S* gene perturbations to get beneficial resistance traits while minimizing pleiotropic effects.

**Figure 3:**
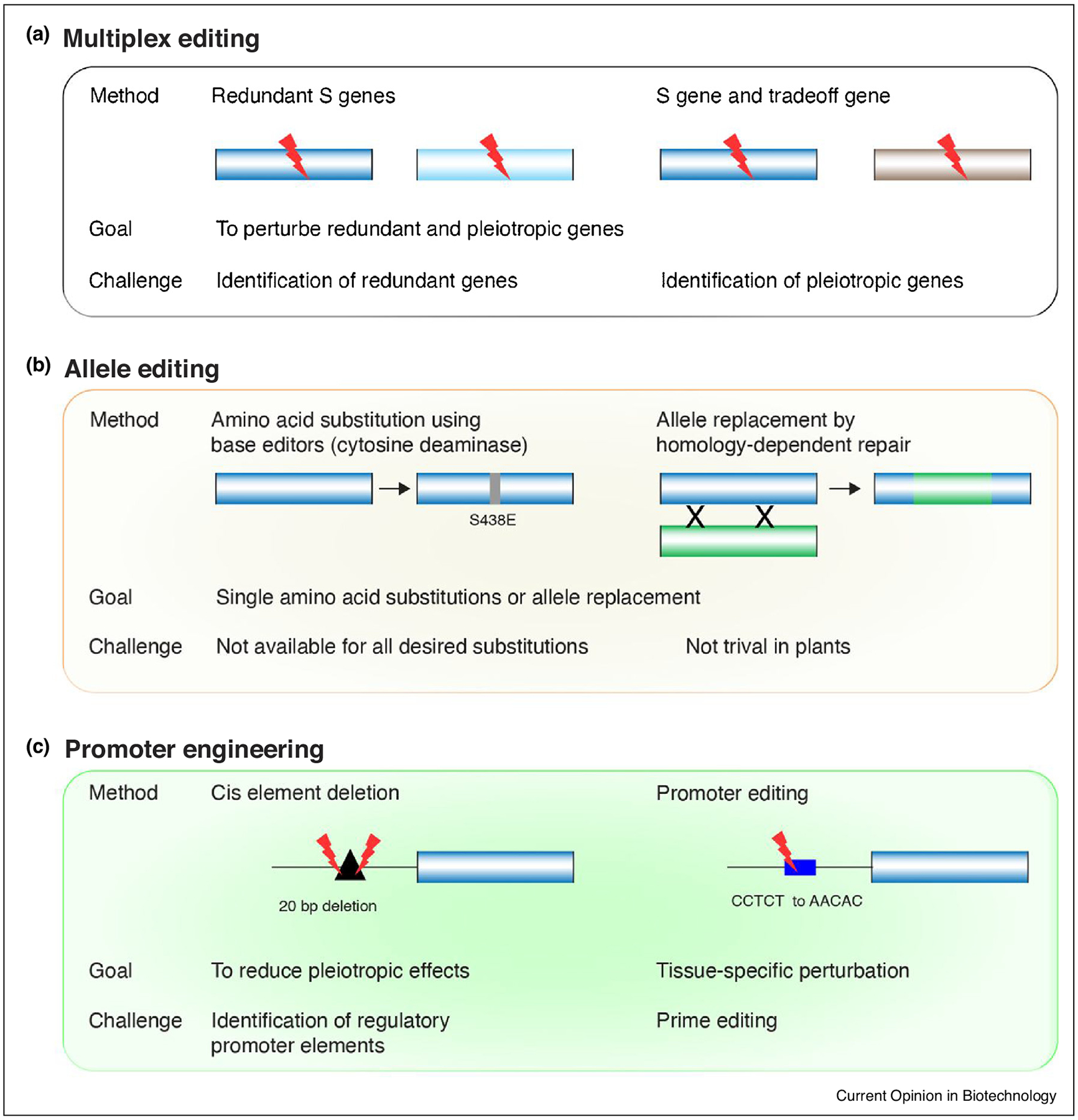
Biotechnological approaches to engineer S genes for pathogen resistance and reduce tradeoff effects. For each approach two methods are illustrated. The goal and challenges are indicated below the diagram. Cylinders indicated open reading frames. Edited areas are marked with a red discharge symbol. **(a)** Multiplex gene editing allows modifications of multiple genes simultaneously, either redundant genes that are members of a gene family, or a combination of an S gene and a tradeoff gene to reduce pleiotropic effects. **(b)** Allele editing might be used to generate hypomorphic alleles that reduce susceptibility but have no or reduced pleiotropic effects. This can be achieved using base editors to make single nucleotide changes and resulting amino acid substitutions, or by replacing larger sequences through homologous recombination. **(c)** Promoters can be engineered to reduce their activity to certain conditions or in specific tissues. Using two guide RNAs the CRISPR/Cas9 system can be used to make precise deletions of selected cis elements in the S gene promoter. Editing of promoter sequences can be achieved using prime editing or homologous recombination.

Multiplex editing is particularly useful for redundant genes, and in hybrid and polyploid genomes (Figure 3a). A technical advance in 2014 was the inactivation of all three *MLO* homoeoalleles in hexaploid wheat [44]. Because of redundancy it was necessary to mutate the alleles in all three subgenomes to achieve powdery mildew resistance. A similar approach was used to mutate *EDR1* in wheat [25]. A powerful future application of multiplex editing is the simultaneous inactivation of susceptibility and tradeoff genes. The bottleneck there is to identify the tradeoff genes that when knocked out reduce pleiotropy without resulting in yet other tradeoffs.

Other ways to reduce pleiotropic effects is by creating or selecting hypomorphic *S* alleles conferring effective loss of susceptibility but with minimized tradeoffs (Figure 3b). These can be generated by base editing [45], as done in *A. thaliana* to introduce non-synonymous substitutions in eIF4E1 to engineer resistance to potyviruses without compromising plant growth [46^•^]. In a similar way, base-editing of eIF4E1 rendered *A. thaliana* resistant to TuMV, while a gene knock-out was hypersusceptible [42]. However, it is not always possible to uncouple growth and immunity, for example, different mutant alleles of the Arabidopsis *JOX2* gene with strong loss of *S* gene function also conferred reduced growth phenotypes [47].

An alternative future advance is to engineer *S* gene promoters to have tissue-specific or condition-specific loss of function to reduce pleiotropy (Figure 3c). Although this is under development, promoter mutations have already been successfully deployed to make *SWEET* genes insensitive to their activation by *Xanthomonas* TAL effectors [16^••^].

The approaches described above would greatly benefit from efficient homologous recombination and prime editing methods that need further technological improvement in plants to be able to edit larger parts within genes or promoters [48].

## Conclusion

Current and future agriculture has to deal with a strong reduction in chemical crop protection, as well as with high disease pressure and new emerging diseases, amongst others, due to climate change. Engineering of *S* genes is a promising method to reduce plant disease and make agriculture more sustainable. To circumvent tradeoffs, it is important to understand the roles of *S* genes in physiology and development. The possibilities of advanced genome editing approaches are manifold, but their use is still restricted in many countries due to regulation of genetically modified organisms. Deregulation would enable the wide utilization of *S* genes for resilient crops that, combined with improved farming practices, ecological principles, and microbiome advances, would contribute to sustainable agriculture.

## Box 1 *mlo*-based and *eif4*-based resistance: conserved and broadly applicable

Two genes stand out when it comes to the broad application of *S* genes in crops, *Mlo* [20] and *eIF4* [51]. Barley plants with *mlo*-based resistance to powdery mildew were discovered in the 1930s and 40s and later on described to be homozygous for recessive alleles of *Mildew resistance* locus O. *Mlo* encodes an integral membrane protein with seven transmembrane domains and is co-expressed with plant defense genes suggesting it plays a role in immunity. However, the molecular function of the MLO protein is still enigmatic. Interestingly, land plants species generally contain multiple *Mlo* genes of which specific clade members can function as *S* genes, as has been shown in a broad range of plant species [20]. In Arabidopsis, three *MLO* genes need to be knocked out (KO) to obtain the strongest resistance to powdery mildew. *mlo*-based resistance by KO or silencing was shown in a dozen of crop species, including tomato, pea and grape. A technological highlight was the generation mildew-resistant *mlo* lines in hexaploid wheat by genome editing [44] or TILLING [52].

A broadly conserved S gene required for potyviruses is *eIF4*, and variants thereof, encoding for eukaryotic translation initiation factor required for mRNA cap recognition and initiation of translation [51]. Translation of potyviral RNA requires recognition by *eIF4* of the viral genome-linked protein (VPg) that caps the 5^′^ end of the viral RNA genome. Recessive resistance to potyviruses, identified in number of plant species, is caused by amino acid substitutions or loss-of-function mutations in eIF4 proteins, which reduce translation of viral RNA. In crops without natural alleles, mutations in eIF4 genes have been selected or generated by genome editing and resistant varieties have thus been engineered.
